# Construction and Performance Evaluation of a Bifidobacterium Delivery System Based on TBP-SA Pickering Emulsion Gels

**DOI:** 10.3390/foods15193502

**Published:** 2026-09-30

**Authors:** Xia Xu, Zhiqiang Yang, Xueli Shen, Yanan Cao, Wei Li, Lianxin Peng, Dingtao Wu, Zunxi Huang, Qian Zhao, Liang Zou, Yu Song

**Affiliations:** 1 Key Laboratory of Coarse Cereal Processing, Ministry of Agriculture and Rural Affairs, Sichuan Engineering & Technology Research Center of Coarse Cereal Industrialization, School of Food and Biological Engineering, Chengdu University, Chengdu 610106, China; 2 School of Leisure Tourism, Chengdu Agricultural College, Chengdu 611130, China; 3 School of Pharmacy, Chengdu University, Chengdu 610106, China; 4 School of Preclinical Medicine, Chengdu University, Chengdu 610106, China; 5 Key Laboratory of Yunnan for Biomass Energy and Biotechnology of Environment, School of Life Science, Yunnan Normal University, Kunming 650500, China; 6 Department of Hepatobiliary and Pancreatic Surgery, Sichuan Cancer Hospital & Institute, Chengdu 610041, China

**Keywords:** emulsion gel, probiotic delivery, Pickering emulsion, tartary buckwheat protein, sodium alginate, *Bifidobacterium longum*

## Abstract

An emulsion gel (EG-B) was fabricated through in situ calcium crosslinking of Pickering emulsions stabilized by tartary buckwheat protein–sodium alginate (TBP-SA) nanocomplexes for the encapsulation and delivery of *Bifidobacterium longum*. Control samples, including free bacteria (Free-B), Pickering emulsions (PE-B), and oil-free hydrogels (HG-B), were used to assess encapsulation efficiency, microstructure, rheological properties, thermal stability, dilution stability, gastrointestinal resistance, storage stability, and colonic adhesion of EG-B. The EG-B exhibited a dual structure comprising a compact Pickering interfacial membrane and a continuous calcium alginate gel network with solid-like rheology (G′>>G″) and viscosity of approximately 100 Pa·s, achieving 92.66% encapsulation efficiency. Viable counts exceeded 6 log CFU/g after pasteurization (72 °C, 15 s) and reached 6.12 log CFU/g post-simulated gastrointestinal digestion, significantly surpassing those of PE-B (5.12 log CFU/g) and HG-B (4.45 log CFU/g). Furthermore, EG-B displayed the highest viable retention during 14-day storage at 4 °C and 25 °C, along with a greater colonic adhesion efficiency (55.64%) compared to PE-B (47.09%) and HG-B (44.65%). The combination of interfacial stabilization and gel network formation provided by the TBP-SA complex presents a straightforward, food-grade strategy for probiotic delivery.

## 1. Introduction

With the rapid advancement of precision nutrition and the functional food industry, the research and development of probiotic products has transitioned from simple raw material addition to sophisticated strategies involving precise active delivery and targeted intestinal colonization [1]. Probiotics, as functional microorganisms, are highly susceptible to environmental stresses, making them vulnerable to inactivation due to temperature fluctuations, pH changes, oxygen partial pressure, and digestive enzymes during food processing, storage, and gastrointestinal digestion. The low viable survival rate of probiotics presents a significant bottleneck that limits their physiological functions and commercial applications [2,3]. Encapsulation and delivery technologies are recognized as effective strategies for enhancing the survival stability of probiotics [4]. Consequently, the development of safe, stable, and efficient probiotic delivery carriers that protect probiotic activity while achieving targeted intestinal delivery under complex conditions has emerged as a key research focus in food biomaterials and functional foods [5,6].

Emulsion gels combine the excellent interfacial stability of Pickering emulsions with the three-dimensional network structure of hydrogels, offering considerable potential for the encapsulation, sustained release, and stress-resistant delivery of bioactive components [4,7,8]. This dual composite structure enhances the physical stability of carriers while establishing multiple protective barriers for encapsulated active substances [8,9,10]. Previous studies successfully fabricated stable tartary buckwheat protein–sodium alginate (TBP-SA) nanocomplexes and structurally robust Pickering emulsions, providing a reliable interfacial foundation for subsequent probiotic encapsulation and delivery [11].

In contrast to this previous work, which focused solely on the fabrication of TBP-SA-stabilized Pickering emulsions and their pH stability, the present study converted the same TBP-SA complex into a structurally integrated emulsion gel (EG-B) via in situ calcium cross-linking (CaCO_3_–GDL) and applied it for probiotic delivery. This gelation strategy was designed to further enhance the structural stability and stress resistance of the carrier system, mitigating probiotic inactivation during processing, storage, and gastrointestinal digestion. To the best of our knowledge, this is the first report of TBP-SA emulsion gels for probiotic encapsulation. Unlike most reported probiotic emulsion gel systems that require an additional separate gelling agent (e.g., xanthan gum [12], gellan gum [13]) to form the gel network, the TBP-SA complex in this system serves simultaneously as the Pickering stabilizer and the gel network precursor, requiring no additional gelling agent, with calcium ions acting only as a cross-linking trigger, thus offering a simpler preparation with entirely food-grade ingredients. In contrast to conventional alginate hydrogels, which rely solely on a cross-linked network without an interfacial barrier, EG-B integrates both a Pickering interfacial membrane and a gel network within a single TBP-SA complex. However, whether such an integrated structure can provide superior protection compared with single-structured carriers remains to be verified. Accordingly, we hypothesized that constructing both the Pickering interfacial membrane and the calcium cross-linked gel network from the same TBP-SA complex would create structural continuity between the interface and the network in EG-B, thereby conferring superior thermal and dilution stability, gastrointestinal tolerance, and storage stability compared with either PE-B (Pickering emulsion alone) or HG-B (hydrogel alone). To test this hypothesis, the protective and delivery performance of EG-B was systematically evaluated and compared with that of PE-B, HG-B, and free bacteria.

In this study, these evaluations covered eight aspects: encapsulation efficiency, microstructure, rheological properties, dilution stability, thermal tolerance, storage stability, gastrointestinal resistance, and colonic mucosal adhesion capacity. The goal of this study is to develop a green and efficient probiotic delivery system based on natural food-grade materials, advancing probiotic applications from simple supplementation to precise, targeted delivery.

## 2. Materials and Methods

### 2.1. Materials

*B. longum* BBMN68 (viable count ≥ 1.0 × 10^10^ CFU/g) was purchased from Shanxi Yuzhou Biotechnology Co., Ltd. (Xi’an, China) as a pure bacterial powder without added protectants and used directly without activation. TBP-SA nanocomplexes were produced in-house. Medium-chain triglycerides (MCT, food-grade) were acquired from Shanghai Yuanye Bio-Technology Co., Ltd. (Shanghai, China). Glucono-δ-lactone (GDL, analytical reagent) and calcium carbonate (CaCO_3_, analytical reagent, ≥99%, SKU: C111980) were obtained from Aladdin Biochemical Technology Co., Ltd. (Shanghai, China).

### 2.2. Preparation of TBP-SA Nanocomplexes and Bifidobacterium Longum-Loaded Carriers

All sample preparation operations took place in a sterile laminar-flow hood. Glassware, centrifuge tubes, pipette tips, and reaction solutions underwent sterilization by autoclaving at 121 °C for 15 min. The rotor and stator of the high-speed homogenizer were disinfected with 70% ethanol, rinsed repeatedly with sterile water, and strict aseptic techniques were upheld throughout to prevent microbial contamination.

#### 2.2.1. Preparation of TBP-SA Nanocomplexes

TBP-SA nanocomplexes were prepared following our previously reported method [11]. Briefly, equal volumes of TBP (2%, *w*/*v*, in pH 7.0 PBS) and SA (0.4%, *w*/*v*, in deionized water) stock solutions were mixed, and the pH of the mixture was adjusted to 7.0. After magnetic stirring for 30 min followed by ultrasonication, the resulting complexes were freeze-dried. Particle size and zeta potential were measured using a Malvern Nano-ZS analyzer (Malvern Instruments Ltd., Malvern, UK). The TBP-SA complexes prepared at pH 7.0 exhibited a hydrodynamic diameter of 166.5 ± 21.9 nm and a zeta potential of −36.53 ± 1.03 mV. Although pH 9.0 was previously identified as optimal for complex formation [11], pH 7.0 was selected here to match the neutral conditions of the probiotic delivery system. The complexes nevertheless retained a small particle size and a highly negative surface charge, consistent with the pH-dependent trends reported previously [11].

#### 2.2.2. Preparation of *Bifidobacterium longum*-Loaded Pickering Emulsions

*B. longum* powder was precisely measured and uniformly dispersed in the MCT oil phase at a concentration of 1% (*w*/*v*) under stirring to serve as the oil phase. Although *B. longum* is hydrophilic and does not dissolve in oil, dispersing the cells in the oil phase prior to emulsification served two purposes: (i) to ensure uniform distribution of the bacteria during emulsion preparation, and (ii) to provide a hydrophobic microenvironment that may reduce oxygen diffusion and help protect the strictly anaerobic bacteria during processing. The viable counts before and after homogenization were both approximately 8 log CFU/mL, indicating that the high-speed shearing step caused no detectable reduction in bacterial viability. TBP-SA lyophilized powder was dissolved in sterile 0.01 M PBS buffer (pH 7.0) to create a 5 wt% TBP-SA aqueous solution. After 30 min of magnetic stirring, the pH was adjusted to 7.0 to form the aqueous phase. The oil and aqueous phases were combined at an oil volume fraction of 60%, followed by high-speed shearing at 12,000 rpm and 4 °C for 2 min. The resulting *B. longum*-loaded Pickering emulsion was designated as PE-B [11].

#### 2.2.3. Preparation of *Bifidobacterium longum*-Loaded Emulsion Gels

Preparation of emulsion gels: Emulsion gels were prepared based on PE-B Pickering emulsions. GDL and CaCO_3_ powders were separately weighed and incorporated into freshly prepared PE-B, achieving a final concentration of 0.25% (*w*/*v*) for each component. The mixture was homogenized at 4000 rpm for 30 s to ensure homogeneous dispersion, then allowed to stand at 4 °C for 12 h to form emulsion gel, referred to as EG-B [13,14].

Preparation of oil-free hydrogels (HG-B): GDL and CaCO_3_ powders were measured and added to a 5 wt% TBP-SA aqueous solution to reach a final concentration of 0.25% (*w*/*v*) for each reagent, alongside the incorporation of 1% (*w*/*v*) *B. longum*. After thorough vortex mixing, the mixture was sheared at 4000 rpm for 30 s and left at 4 °C for 12 h to complete cross-linking, resulting in the oil-free hydrogel labeled HG-B.

### 2.3. Enumeration of Viable Probiotic Cells

Viable bacterial counts in the samples were determined using the plate-counting method [14,15]. Briefly, 1 g of gel samples was accurately weighed, or 1 mL of liquid samples was pipetted, and mixed with a suitable volume of sterile physiological saline to create an initial 1:10 dilution. Serial 10-fold dilutions were then performed. Following this, 100 μL of the appropriately diluted bacterial suspension was evenly spread onto MRS agar medium. The spread plates were inverted and placed in anaerobic culture bags containing anaerobic indicators to monitor anaerobic conditions. After sealing, the bags were incubated in a CO_2_ incubator at 37 °C for 48 h. Plates containing 30–300 colonies were selected for counting. Results were expressed as CFU/mL for liquid samples (e.g., the bacterial suspension in the oil phase and the Pickering emulsion) and as CFU/g for semi-solid gel samples (EG-B and HG-B), reflecting the sampling form of each matrix.

### 2.4. Determination of Encapsulation Efficiency

Encapsulation efficiency was assessed via the plate-counting method according to established protocols [16]. Briefly, approximately 3 g of freshly prepared samples (PE-B, EG-B, HG-B) were centrifuged at 5000 rpm for 10 min at 4 °C to separate unencapsulated free bacterial cells. The resulting precipitate containing free cells was collected and resuspended in 0.85% sterile physiological saline to prepare a bacterial suspension, which was then plated, incubated, and counted for free viable cells. Encapsulation efficiency was calculated using the following formula:

$$E(\%)\;=\;\frac{{N-N}_{1}}{N}\times 100$$

where *N* represents the total viable count before emulsification/encapsulation and *N*_1_ represents the count of unencapsulated (leaked) viable cells, both expressed in consistent units for each sample type (CFU/mL for PE-B and CFU/g for the gel samples).

### 2.5. Microstructural Characterization

(1) Confocal laser scanning microscopy (CLSM)

Observation of emulsion microstructure [17]: Test samples underwent a 1:2 dilution with deionized water and were labeled using dual-fluorescence with Nile Red (1 mg/mL) and Fast Green (1 mg/mL). Staining took place in darkness at room temperature for 30 min, where Nile Red targeted the oil phase, and Fast Green labeled the aqueous TBP-SA complex fraction. Subsequent observations were conducted using CLSM (Leica Microsystems (Shanghai) Co., Ltd., Shanghai, China), with excitation/emission wavelengths set at 488 nm/550–600 nm for Nile Red and 633 nm/650–700 nm for Fast Green.

Observation of live/dead bacterial distribution: Fluorescent staining of *B. longum* was performed using a SYTO 9/PI Live/Dead staining kit (Thermo Fisher Scientific, Waltham, MA, USA) according to the manufacturer’s instructions [18,19]. CLSM imaging was conducted with a 40 × objective lens, employing excitation/emission wavelengths of 485 nm/498 nm for SYTO 9 and 535 nm/617 nm for PI.

(2) Cryo-scanning electron microscopy (Cryo-SEM)

Cryo-SEM (Hitachi SU 8100; Hitachi, Ltd., Tokyo, Japan) characterized the ultrastructure of samples. Uniform coating occurred on specimen stubs affixed with conductive carbon adhesive tape, followed by rapid freezing in liquid nitrogen for 30 s. Under low-temperature vacuum conditions, frozen samples were transferred to a pre-treatment chamber for surface sublimation dehydration and gold sputter-coating to enhance electrical conductivity, after which microstructural images were acquired.

### 2.6. Dilution Stability

To simulate pH-dependent dilution conditions in vivo and assess the structural stability of TBP-SA near its isoelectric point, three buffer solutions at pH 2.5, 4.5, and 7.0 were prepared, with pH 4.5 corresponding to the isoelectric point of TBP [11,20]. Samples of PE-B, EG-B, and HG-B were placed at the bottom of transparent glass vials, and the corresponding buffer was gently layered along the vial wall at a 1:1 (*v*/*v*) ratio without agitation. The vials were then left to stand at 25 °C for 6 h. Macroscopic appearance changes, including layer separation and demulsification, were photographed to assess the dilution resistance of the carrier systems.

### 2.7. Rheological Properties

Rheological measurements employed a HAAKE rotational rheometer (Thermo Fisher Scientific, Waltham, MA, USA) with a P60 TiL parallel-plate geometry (smooth polished titanium, 40 mm diameter) [21]. The measurement gap was set at 1 mm, and the sample loading volume was 1.25 mL. The temperature was maintained at 25 ± 0.1 °C. Steady-shear tests were performed with the shear rate linearly increased from 0 to 100 s^−1^, and 60 data points were collected. Subsequently, amplitude sweep tests were conducted from 0.01% to 100% strain at a constant frequency of 1 Hz to determine the linear viscoelastic region (LVR), which was identified as the strain range in which the storage modulus (G′) decreased by less than 10% from its low-strain plateau value; the LVR was determined to be 0.01–1%. Frequency sweep tests were then performed from 0.01 to 100 Hz at a constant strain of 0.1% (within the LVR). Samples were allowed to equilibrate for 3 min after loading, prior to testing, and no pre-shear was applied. Data below 0.1 Hz were excluded from analysis owing to low-torque instrumental noise, and results are therefore presented over the range 0.1–100 Hz. The storage modulus (G′) and loss modulus (G″) were recorded.

### 2.8. Thermal Protection

To simulate thermal processing conditions relevant to the food industry, free probiotics (Free-B) and probiotic-loaded samples (PE-B, EG-B, HG-B) underwent water-bath heating at 72 °C for 15 s or 65 °C for 30 min [14]. Following cooling, viable counts of probiotics post-heat treatment were determined following the procedures outlined in Section 2.3 to evaluate the thermal-protective performance of different carriers.

### 2.9. Gastrointestinal Tolerance

Simulated gastric fluid (SGF) and simulated intestinal fluid (SIF) were prepared with modifications based on the static INFOGEST in vitro digestion model [22,23]. SGF consisted of 10 g/L pepsin and 9 g/L NaCl dissolved in sterile water, with pH adjusted to 1.2 using 1.0 M HCl. SIF included 10 g/L pancreatin, 6.8 g/L KH_2_PO_4_, and 10 g/L bile salts in sterile water, with pH set to 7.0 using 1.0 M NaOH. All digestive fluids underwent filter sterilization through 0.22 μm membrane filters.

Approximately 1.0 g of each test sample (Free-B, PE-B, EG-B, HG-B) was accurately weighed and recorded. Samples were mixed with SGF at a ratio of 1:10 (*w*/*v*) and digested under continuous stirring at 37 °C for 2 h. Upon completion of gastric digestion, the pH was adjusted to 5.0 using 1.0 M NaOH to terminate the reaction. Following this, 10 mL of sterile SIF was added, and the mixture’s pH was adjusted to 7.0, followed by further digestion at 37 °C for 6 h. Samples were collected at 1 and 2 h of gastric digestion and at 2, 4, and 6 h of intestinal digestion for viable count measurement. Macroscopic morphology was documented at the initial time point, the conclusion of gastric digestion, and the end of intestinal digestion. All procedures were conducted within a sterile laminar-flow hood.

### 2.10. Storage Stability

Probiotic-loaded samples (PE-B, EG-B, HG-B) were stored in the dark at 4 °C and 25 °C for 14 days. Samples were collected on day 0, day 7, and day 14, with viable bacterial counts determined using the plate-counting method [24].

### 2.11. Colonic Adhesion Efficiency

An everted mouse colonic sac model was utilized to measure the colonic adhesion efficiency (AE) of the samples [16]. Male KM mice were fasted for 12 h but allowed free access to drinking water prior to experiments. Mice were euthanized via cervical dislocation, after which colon tissues were rapidly excised, rinsed with sterile physiological saline to remove intestinal content, and carefully cleared of surface adipose tissues and blood vessels. Colon segments were then gently everted and immersed in sterile PBS buffer (pH 7.0) at 4 °C for subsequent use.

Digestion-treated carrier systems (PE-B, EG-B, HG-B) and free *B. longum* (Free-B) containing equivalent viable bacteria were diluted with sterile PBS buffer (pH 7.0) to mitigate interference due to differences in initial viable counts among groups in the adhesion assay. Each bacterial suspension was co-incubated with colon segments at 37 °C for 1 h. Afterward, colon segments were washed gently three times with sterile water. Bacteria adhering to colonic mucosa were quantified using a plate-counting assay. AE was calculated using the following formula:

$$\mathrm{AE}(\%)\;=\;\frac{N_{1}}{N}\times 100$$

where *N*_1_ is the number of *B. longum* adhering to colon segments (CFU); *N* is the total initial number of added *B. longum* (CFU).

Cryo-SEM facilitated observation of the surface morphology of colon segments before and after bacterial adhesion. Samples were rapidly frozen in liquid nitrogen, fractured under vacuum, and subjected to sublimation at −95 °C for 4 min. Following platinum sputter-coating, specimens were visualized at an acceleration voltage of 2 kV under −180 °C to visualize bacterial adhesion to mucosal surfaces.

### 2.12. Data Analysis

All experiments were conducted in triplicate, and results were expressed as mean ± standard deviation. Data visualization was performed using Origin 2025 or GraphPad Prism 9, and statistical analysis was conducted using SPSS 27.0. One-way analysis of variance (ANOVA) followed by Tukey’s multiple-comparison test was used for comparisons among multiple groups at each time point. For time-course experiments (simulated gastrointestinal digestion and storage stability), a two-way ANOVA with group and time as factors was performed to assess the main and interaction effects, followed by Tukey’s post hoc test. A *p*-value < 0.05 was considered statistically significant.

## 3. Results and Discussion

### 3.1. Encapsulation Efficiency

**Figure 1:**
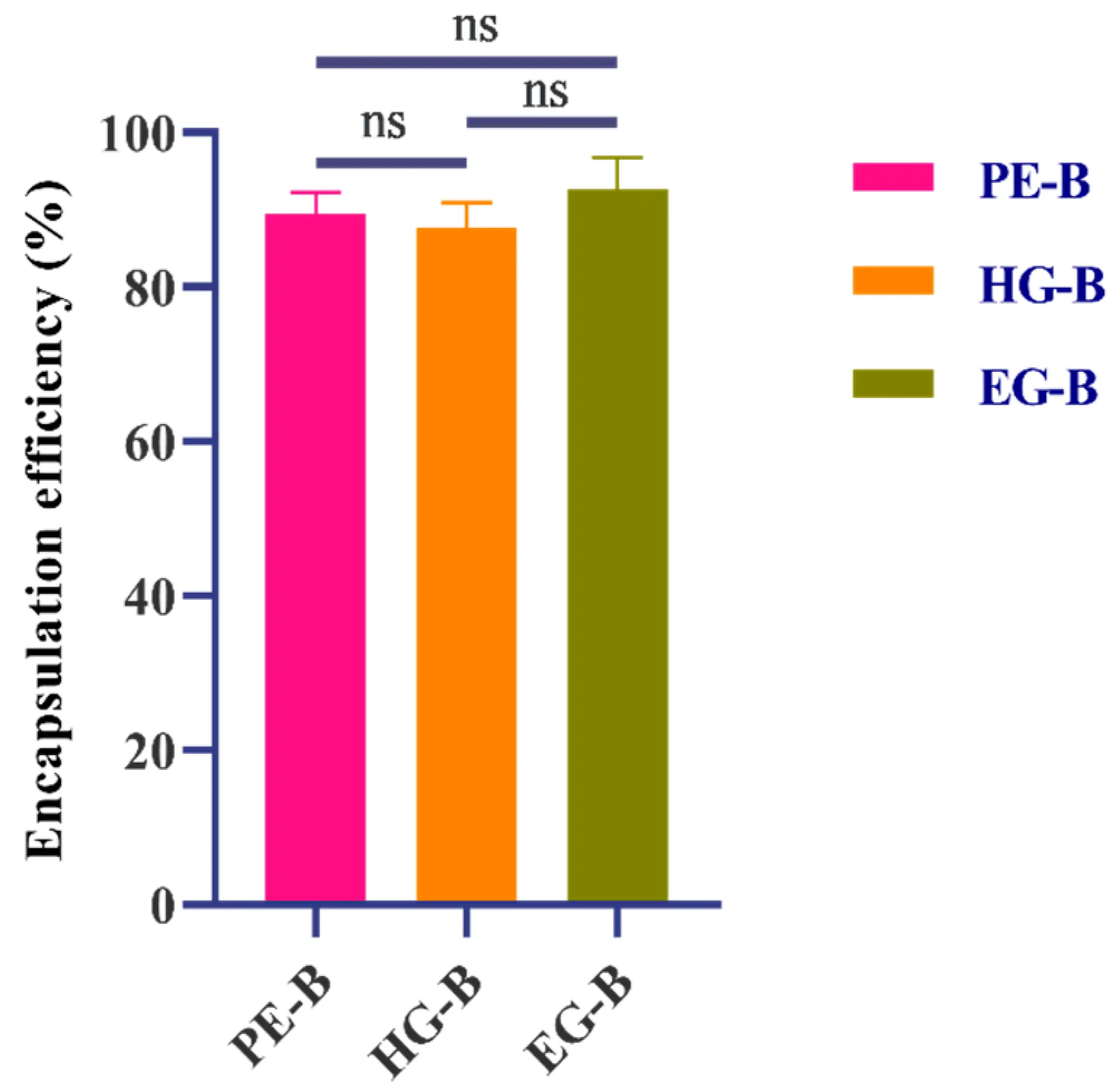
Encapsulation efficiency of *Bifidobacterium longum* in the three delivery systems. Data are presented as mean ± standard deviation (*n* = 3). ns, not significant (*p* > 0.05, Tukey’s multiple-comparison test).

The encapsulation efficiency of various delivery systems for *B. longum* is illustrated in Figure 1. All three delivery systems demonstrated strong encapsulation capacities for *B. longum*, with encapsulation efficiencies of 89.62 ± 2.63%, 87.68 ± 2.54%, and 92.66 ± 3.45% for PE-B, HG-B, and EG-B, respectively. Statistical analysis indicated no significant differences in encapsulation efficiency among the three groups (*p* > 0.05), suggesting that all carrier structures effectively encapsulated *B. longum*.

Despite the lack of significant differences in encapsulation efficiency, the cell entrapment mechanisms varied among the three systems. The high encapsulation efficiency of PE-B was primarily due to the dense interfacial membrane formed by TBP-SA nanocomplexes at the oil–water interface. This robust Pickering interfacial layer effectively minimized bacterial cell leakage during centrifugation or washing procedures [25,26]. In contrast, the encapsulation capacity of HG-B relied mainly on the three-dimensional network pore structure facilitated by calcium cross-linking of SA, providing a reliable physical entrapment space for bacterial cells [25,26,27]. The encapsulation efficiency of EG-B was numerically higher than that of the other groups, suggesting a potential synergistic effect between the interfacial membrane and the gel network. Specifically, in situ gelation preserved the Pickering interfacial layer, while the CaCO_3_–GDL-induced gel network enhanced steric hindrance in the continuous phase. These features collectively restricted bacterial cell diffusion, consistent with this trend [14,16].

These findings highlight that TBP-SA complexes can offer effective physical entrapment, functioning as both interfacial stabilizers and gel matrices. Furthermore, the in situ cross-linking preparation method of emulsion gels did not adversely affect encapsulation efficiency, thus establishing a structural basis for subsequent protection of probiotic cell viability.

### 3.2. Microstructure

**Figure 2:**
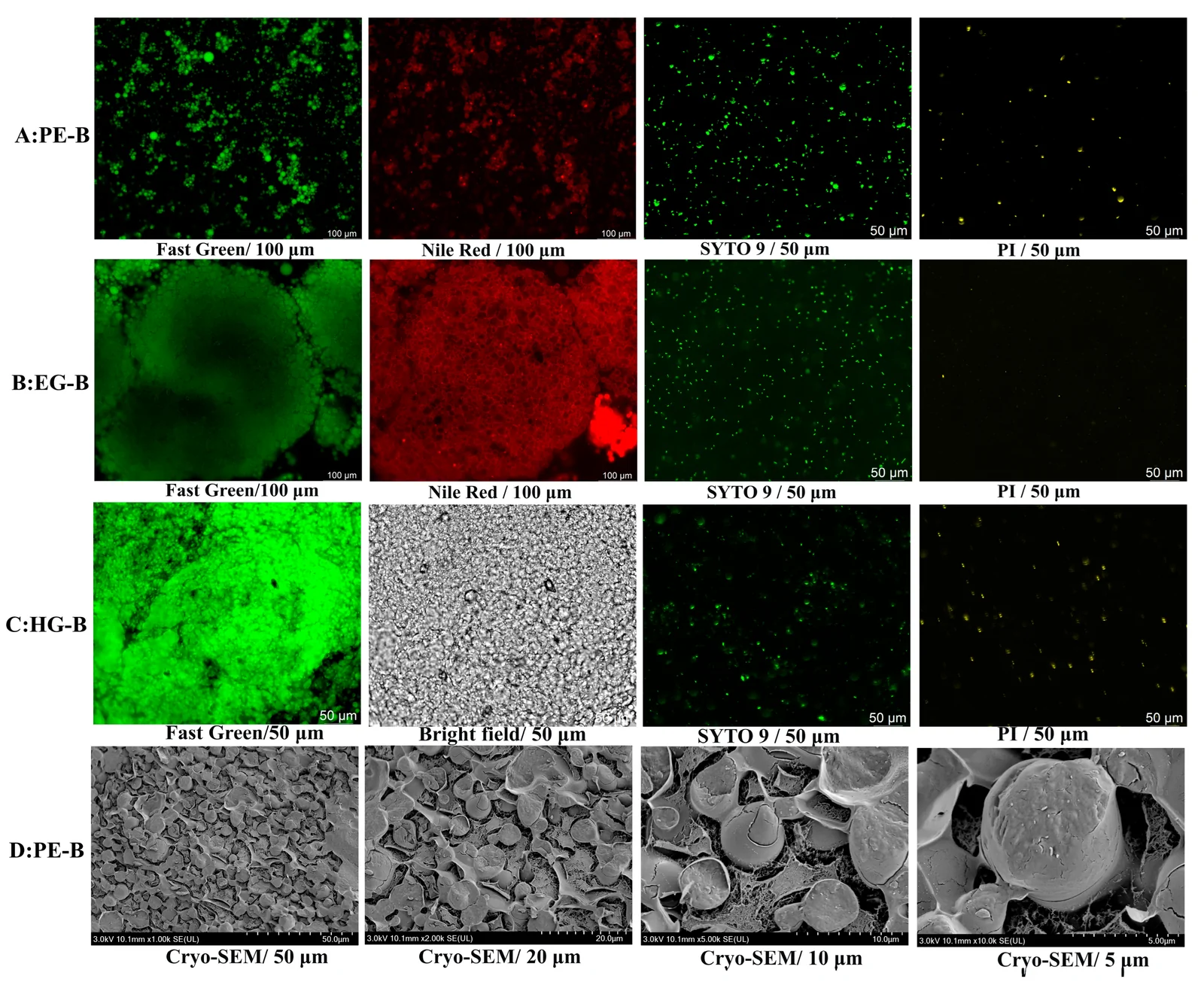
Microstructure of the different delivery systems. (**A**) CLSM image of PE-B (Fast Green/TBP-SA aqueous phase + Nile Red/oil phase + SYTO 9/viable cells + PI/dead cells); (**B**) CLSM image of EG-B (Fast Green/TBP-SA aqueous phase + Nile Red/oil phase + SYTO 9/viable cells + PI/dead cells); (**C**) CLSM image of HG-B (Fast Green/TBP-SA aqueous phase + bright-field + SYTO 9/viable cells + PI/dead cells); (**D**) Cryo-SEM image of PE-B. Green: Fast Green-labeled TBP-SA aqueous phase; red: Nile Red-labeled oil phase; bright green: SYTO 9-labeled viable cells; yellow: PI-labeled dead cells.

To explore the internal microstructure of the EG-B emulsion gel and the encapsulation state of *B. longum*, CLSM and Cryo-SEM were employed for characterization. Results are presented in Figure 2.

(1) CLSM observation

CLSM images of PE-B, EG-B, and HG-B are illustrated in Figure 2A–C. In PE-B, the Fast Green-labeled TBP-SA complex aqueous matrix displayed a diffuse distribution, while the Nile Red-labeled oil phase appeared as uniformly dispersed discrete droplets within the aqueous matrix, confirming a typical oil-in-water emulsion structure [28]. SYTO 9-labeled viable-cell fluorescence was evenly distributed across the field of view, accompanied by a small amount of PI-labeled dead-cell fluorescence. This observation indicates that the fluid emulsion, lacking a cross-linked network, did not provide a rigid physical barrier, rendering *B. longum* vulnerable to inactivation under external environmental stresses [12].

In the EG-B emulsion gel formed through in situ cross-linking with GDL and CaCO_3_, Fast Green fluorescence revealed a large-area continuous cross-linked aqueous network, effectively entrapping red oil droplets within the gel pores. No evidence of free oil-phase separation or droplet coalescence was observed. Abundant viable-cell fluorescence was present throughout the field of view, while PI-labeled dead-cell fluorescence signals were nearly undetectable. This indicates that the combination of the continuous hydrophilic gel skeleton and embedded hydrophobic oil droplets created a composite protective system capable of significantly preserving the viability of *B. longum* [12,14].

The oil-free HG-B hydrogel displayed only continuous and uniform Fast Green fluorescence, with the absence of characteristic Nile Red oil-phase fluorescence. The distribution density of viable cells in this system was comparable to that in EG-B; however, a significant increase in the intensity of PI-labeled dead-cell fluorescence was noted. This finding suggests that the purely aqueous cross-linked network lacked a hydrophobic barrier and was ineffective at preventing oxygen permeation, making the strictly anaerobic *B. longum* vulnerable to oxidative damage [29].

(2) Cryo-SEM observation

Cryo-SEM characterization of PE-B (Figure 2D) provided additional insights into the ultrastructure of the oil–water interface. At low magnification, the system displayed uniform-sized spherical oil droplets that were evenly dispersed, aligning with CLSM findings of oil/water dual staining. High-magnification imaging showed that individual oil droplets were fully enveloped by a dense TBP-SA-particle interfacial membrane without noticeable defects, representing typical Pickering interfacial characteristics. This intact interfacial layer can persist during in situ gelation and serves as a fundamental component of the composite structure of EG-B.

Comprehensive characterization results indicated that PE-B featured a single oil–water interfacial barrier, HG-B consisted solely of a pure hydrogel network, while EG-B exhibited a dual architecture comprising both a Pickering interfacial membrane and a three-dimensional cross-linked gel network. This dual structure provided multi-level protection for *B. longum*, with the oil–water interface entrapping bacterial cells and insulating them from gastric acid, while the gel network endowed the system with dilution resistance, shear tolerance, and potential oxygen-barrier capacity. Collectively, these components may help reduce heat conduction and bile-salt permeation, thereby contributing to resistance against various environmental stresses [14]. Such structure–function relationships resulting from interfacial-gel synergy are documented in comparable composite emulsion gel systems [30,31]. The microstructural differences among the three carriers offer both structural and theoretical support for subsequent functional evaluations, including gastrointestinal tolerance and storage stability.

### 3.3. Dilution Stability Analysis

**Figure 3:**
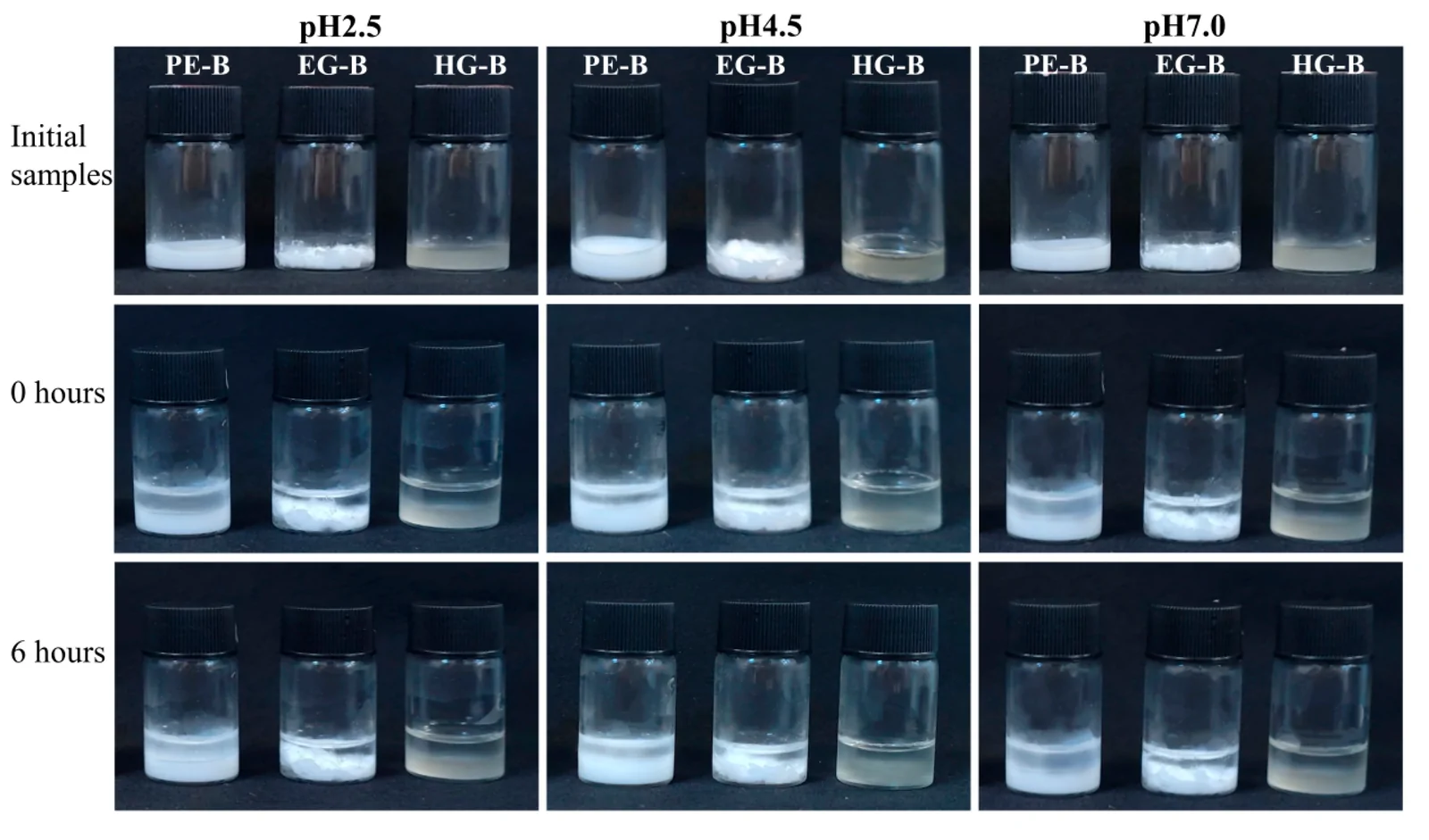
Macroscopic morphology illustrating the dilution stability of PE-B, EG-B, and HG-B under different pH conditions. The boundary observed is due to the static layering method and the density difference between the sample and the buffer, rather than phase separation.

The macroscopic stability results of samples subjected to different pH-dilution conditions are depicted in Figure 3. PE-B maintained a uniform appearance across all pH conditions, with no noticeable phase separation, indicating that the Pickering emulsion demonstrated a degree of tolerance to dilution. Both EG-B and HG-B retained their intact gel morphology under the three pH conditions, exhibiting no signs of structural disintegration or oil–water separation. The structural integrity provided by gelation was the main factor contributing to their dilution stability; specifically, the calcium-alginate cross-linked network served as a physical scaffold that effectively resisted structural disruption induced by dilution [32]. The performance of EG-B and HG-B was comparable across all pH conditions, suggesting that the presence of the oil phase did not negatively impact the system’s dilution stability, with the gel cross-linked network being the fundamental structure governing this property [32,33].

The boundary observed immediately after addition is a result of the static layering method and the density difference between the sample and the buffer, rather than phase separation. The persistence of this boundary after 6 h indicates that the gel network remained intact and resisted dilution. Note that CaCO_3_ serves only as a Ca^2+^ source during gelation and is largely consumed during the 12 h cross-linking period; therefore, little residual CaCO_3_ is available to dissolve upon subsequent acid exposure. In addition, calcium-alginate gels are known to shrink and become denser under acidic conditions rather than disintegrating [34], which explains the comparable stability of EG-B and HG-B observed at pH 2.5 and pH 7.0.

### 3.4. Rheological Properties Analysis

**Figure 4:**
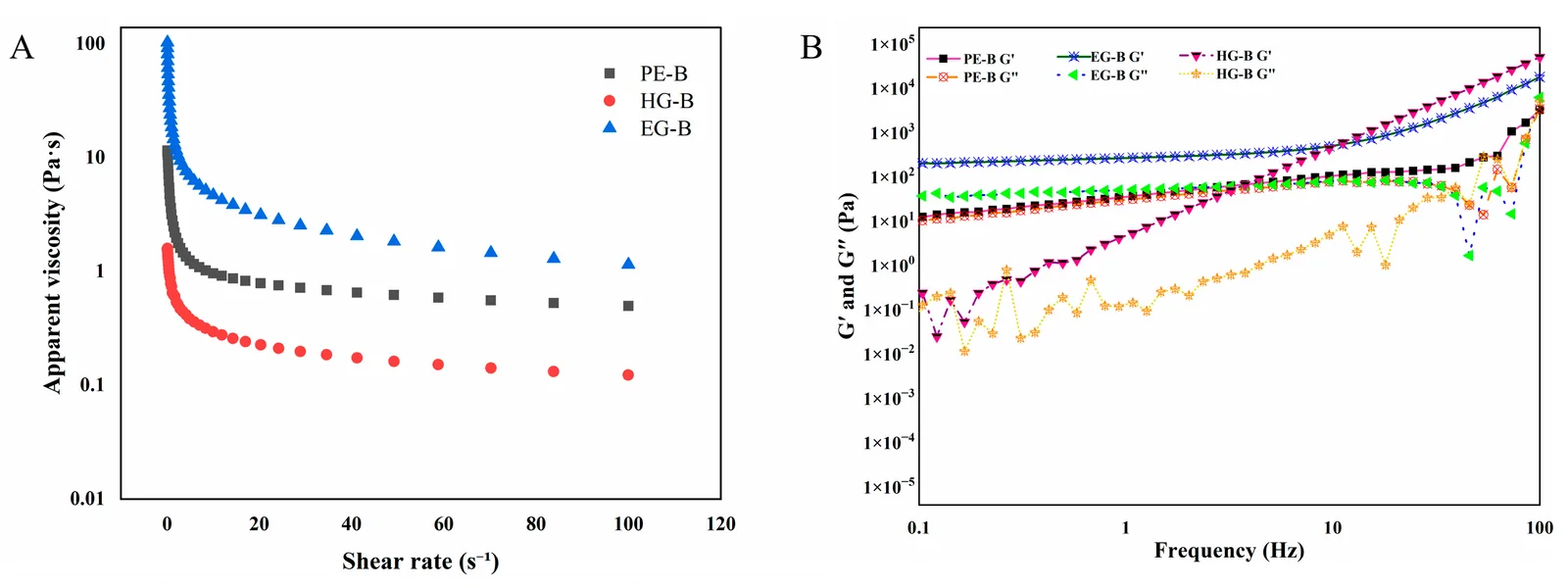
(**A**) Apparent viscosity of PE-B, HG-B, and EG-B as a function of shear rate; (**B**) Storage modulus G′ and loss modulus G″ as a function of frequency for PE-B, HG-B, and EG-B. Data are presented as mean ± standard deviation (*n* = 3).

The variation in apparent viscosity as a function of shear rate for the different systems is shown in Figure 4A. All three samples demonstrated typical shear-thinning behavior, whereby apparent viscosity decreased continuously with increasing shear rate, aligning with the rheological characteristics of food-gel and emulsion systems [35,36]. Throughout the entire shear-rate range, the apparent viscosity followed the order: EG-B > PE-B > HG-B. Frequency sweep tests (Figure 4B) further revealed that EG-B maintained G′ values substantially higher than G″ (G′≫G″) across the entire frequency range, confirming solid-like gel behavior. HG-B and PE-B displayed moduli that increased with frequency, with HG-B showing very low G′ at low frequencies. The differences in rheological properties among the three systems corresponded well with their microstructural characteristics.

Viscosity differences reflected the microstructural attributes of each system. The viscosity of PE-B was primarily attributed to the close packing of oil droplets resulting from its high oil volume fraction (60%), along with the hindering effect of the TBP-SA particle-stabilized interfacial membrane, which provided mechanical strength against relative motion between droplets [37,38]. HG-B relied exclusively on the calcium-alginate cross-linked network for flow resistance, leading to the lowest viscosity. In contrast, the apparent viscosity of EG-B reached approximately 100 Pa·s at low shear rates, significantly exceeding that of the other two systems. This observation was consistent with Cryo-SEM findings of closely packed oil droplets and an interpenetrating continuous gel-skeleton architecture, which collectively enhanced the flow resistance of the system.

From an application perspective, EG-B achieved high viscosity at low shear rates, effectively inhibiting oil-droplet sedimentation and bacterial-cell aggregation during storage, thereby enhancing the static stability of the system. Furthermore, the substantial drop in viscosity under high-shear conditions conferred favorable shear adaptability, making it suitable for industrial pumping and filling, as well as the dynamic shear environment generated by gastrointestinal peristalsis. Elevated viscosity at low shear rates also increased matrix diffusion resistance, retarding the permeation rates of H^+^, pepsin, and bile salts [39] and thus providing long-term protection for probiotics. These observations were consistent with subsequent gastrointestinal tolerance results.

### 3.5. Thermal Protection Analysis

**Figure 5:**
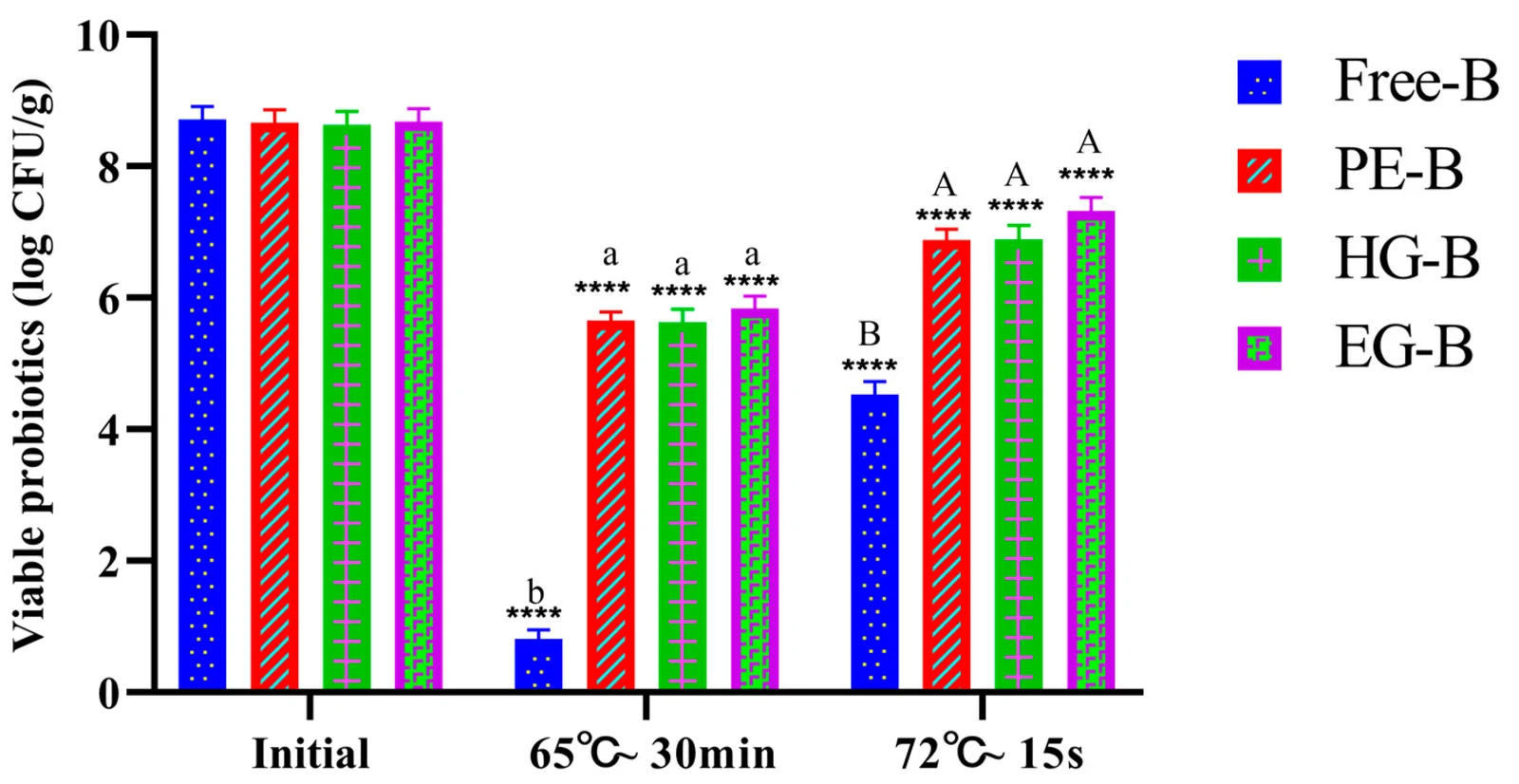
Thermal protective effects of different delivery systems on *B. longum*. **** indicates a highly significant difference compared to the corresponding initial group (*p* < 0.0001). Different lowercase letters denote significant differences among samples within the 65 °C treatment group, while different uppercase letters indicate significant differences among samples within the 72 °C treatment group (Tukey’s test, *p* < 0.05). Data are presented as mean ± standard deviation (*n* = 3).

The thermal protective effects of various delivery systems are illustrated in Figure 5. Following treatment at 65 °C for 30 min, the viable count of Free-B decreased dramatically to 0.81 log CFU/g, indicating nearly complete inactivation. In contrast, the viable counts for PE-B, HG-B, and EG-B were sustained at 5.65, 5.63, and 5.84 log CFU/g, respectively, all significantly higher than that of Free-B (*p* < 0.0001). However, no significant differences were detected among the three encapsulated systems (*p* > 0.05). After exposure to 72 °C for 15 s, the viable count of Free-B was recorded at 4.53 log CFU/g, whereas PE-B, HG-B, and EG-B maintained viable counts of 6.88, 6.89, and 7.32 log CFU/g, respectively. Again, these counts were highly significantly higher than that of Free-B (*p* < 0.0001), with no significant differences among the three encapsulated systems (*p* > 0.05). Although not statistically significant, the mean viable counts of EG-B were slightly higher than those of PE-B and HG-B during both heat-treatment conditions, suggesting a trend of superior protection.

From an industrial application standpoint, the 72 °C for 15 s treatment simulates a typical HTST pasteurization process. After this treatment, the viable counts of PE-B, HG-B, and EG-B all exceeded 6 log CFU/g, which aligns with the generally recommended viable-cell threshold for probiotic products. Under sustained heat treatment at 65 °C, all three systems maintained viable counts above 105 CFU/g, representing a substantial 4.8-log cycle enhancement compared to Free-B. This protective effect correlates well with their composite structural advantages: the dispersed oil phase, with its relatively low thermal conductivity, may extend the heat-transfer path and slow heat transfer [40,41]. In addition, the abundant water trapped within the gel network could absorb some heat and buffer temperature increases, potentially mitigating heat-induced protein denaturation and cellular damage in probiotic cells [42,43]. Under the moderate heat-treatment conditions applied in this study, the gel network’s physical-barrier effect was predominant, while the contribution from the oil phase was marginal, which may explain the comparable protective performance among the three carrier systems. It is anticipated that the composite structural benefits of EG-B would be more pronounced under harsher heat-stress conditions.

In summary, all three TBP-SA-based carriers substantially improved the survival of *B. longum* under both heat-treatment scenarios. Viable counts remained above 6 log CFU/g following HTST pasteurization, demonstrating favorable adaptability to food-processing applications.

### 3.6. Gastrointestinal Tolerance Analysis

**Figure 6:**
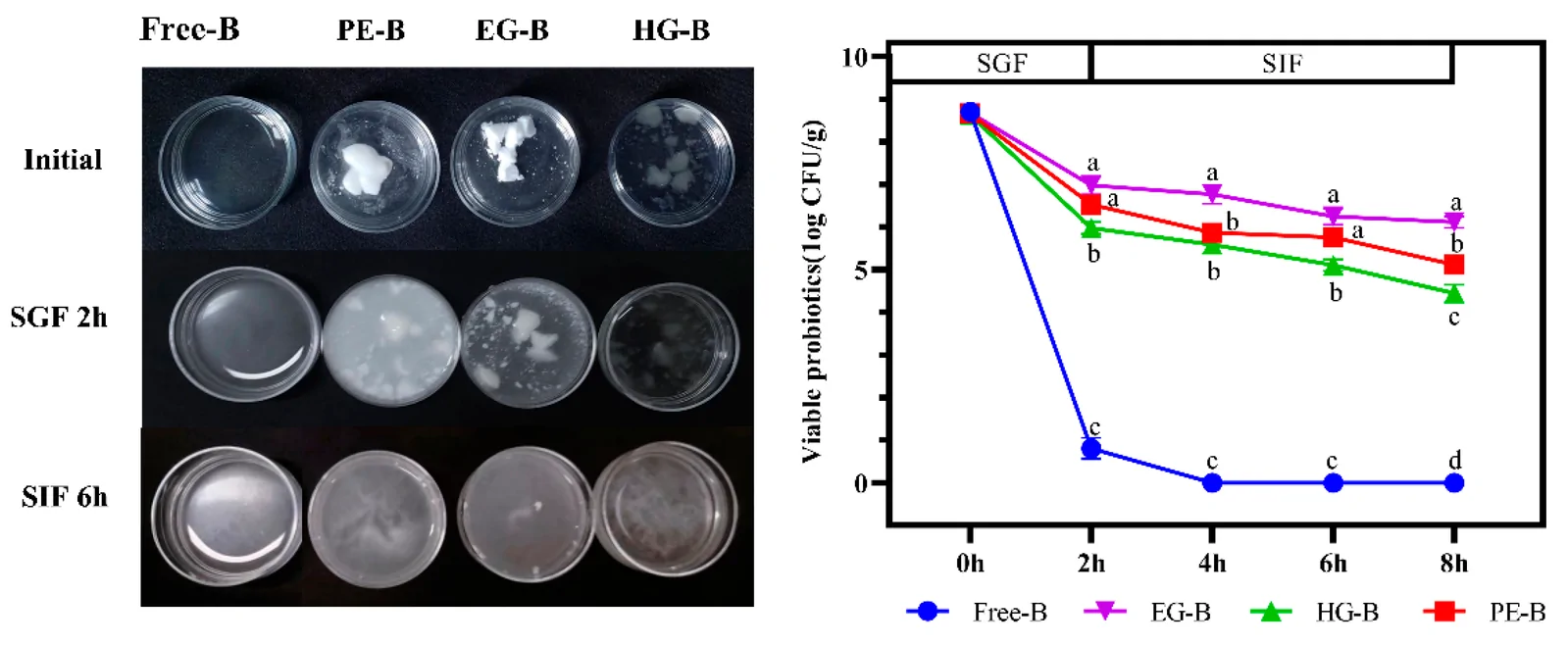
Viable-count changes in *Bifidobacterium longum* in different delivery systems during simulated gastrointestinal digestion. Different lowercase letters at the same time point indicate significant differences among groups (Tukey’s multiple-comparison test, *p* < 0.05). Data are presented as mean ± standard deviation (*n* = 3).

The dynamic changes in viable counts of probiotics across each group during simulated gastrointestinal digestion are depicted in Figure 6. Following 2 h of SGF digestion, the viable count of free probiotics (Free-B) dropped sharply to 0.81 log CFU/g. In contrast, PE-B and EG-B exhibited the highest survival rates, maintaining viable counts of 6.53 and 6.98 log CFU/g, respectively, with no significant difference observed between these two groups (*p* > 0.05). The viable count for HG-B was recorded at 5.98 log CFU/g, which was significantly lower than those of PE-B and EG-B (*p* < 0.05), but still markedly higher than that of Free-B (*p* < 0.0001).

As the samples transitioned into the SIF intestinal digestion phase, viable counts for all groups continued to decline gradually due to multiple stresses, including bile-salt erosion, pancreatin hydrolysis, and dynamic stirring. Free-B was completely inactivated after 2 h of intestinal digestion, while the encapsulated groups maintained bacterial viability throughout the digestion period. At the endpoint of the total 8 h digestion, EG-B displayed the highest viable count (6.12 log CFU/g), followed by PE-B (5.12 log CFU/g), and HG-B yielded the lowest value at 4.45 log CFU/g. Significant differences were observed among the three groups (*p* < 0.05). A two-way ANOVA revealed significant main effects of group and time, as well as a significant group × time interaction, on viability (all *p* < 0.001), highlighting a clear hierarchy in protective efficacy.

Integrating the preceding stability results elucidates the differences in protective mechanisms among these systems. The pure gel network of HG-B effectively tolerated static pH dilution but exhibited poor resistance against dynamic bile-salt and enzyme-mediated erosion. While the single interfacial membrane of PE-B could withstand gastric-acid stress, exposure to bile salts and dynamic stirring in intestinal fluid may have weakened its interfacial barrier, leading to a gradual reduction in protective efficacy [44,45]. Conversely, EG-B, characterized by a dual architecture of a Pickering interfacial membrane and a gel network, simultaneously resisted multiple damaging factors induced by gastric acid, bile salts, digestive enzymes, and dynamic stirring, thereby achieving long-term protection throughout the entire digestion cycle. These findings suggest that the two evaluation models—static dilution-stability tests and dynamic digestion assays—complementarily characterize carrier structural stability.

### 3.7. Storage Stability Analysis

**Figure 7:**
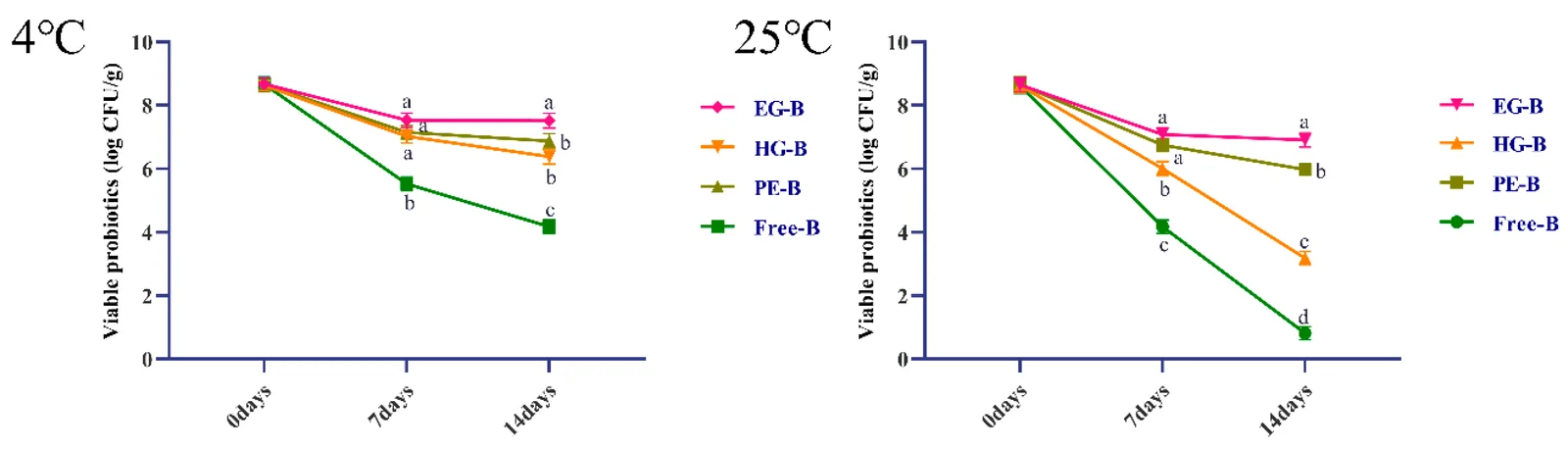
Viable-count changes in *Bifidobacterium longum* in different delivery systems during storage at 4 °C and 25 °C. Different lowercase letters at the same time point denote significant differences among groups (Tukey’s multiple-comparison test, *p* < 0.05). Data are presented as mean ± standard deviation (*n* = 3).

The survival results of probiotics stored at 4 °C and 25 °C in the dark for 14 days are presented in Figure 7. At 4 °C, no significant differences in viable counts were found among the three carrier systems on day 7, with all displaying significantly higher counts than free bacteria. After 14 days, the viable count of EG-B remained elevated at 7.52 log CFU/g, significantly surpassing those of PE-B (6.88 log CFU/g) and HG-B (6.39 log CFU/g), thereby demonstrating optimal long-term protective effects under low-temperature conditions.

At 25 °C, temperature-related stress increased. On day 7, viable counts for EG-B and PE-B were 7.09 and 6.75 log CFU/g, respectively, significantly higher than HG-B (6.01 log CFU/g) and Free-B (4.18 log CFU/g) (*p* < 0.05). By day 14, EG-B exhibited the highest viable count (6.91 log CFU/g), followed by PE-B (5.98 log CFU/g). In contrast, HG-B declined to 3.19 log CFU/g, while Free-B approached near-complete inactivation (0.82 log CFU/g). Significant differences were observed among the four groups (*p* < 0.05). Similarly, two-way ANOVAs (group × time) at 4 °C and 25 °C each revealed significant main effects of group and time, as well as a significant group × time interaction (all *p* < 0.001).

The interfacial layer of PE-B may help mitigate oxygen-induced damage to bacterial cells at low temperatures; however, the protective capacity of this single interfacial barrier appeared to diminish at higher temperatures. HG-B experienced the most severe decline in viable counts under both conditions, suggesting that the oil-phase-free gel network has a relatively limited oxygen-blocking capacity. The combined oil-droplet-gel-network structure of EG-B may provide both oxygen-barrier and physical-encapsulation functions, contributing to its optimal long-term storage protection under both temperature conditions. This system effectively addressed the challenge of probiotic inactivation during storage, markedly enhancing product shelf-life and offering significant potential for industrial applications.

### 3.8. Colonic Adhesion Efficiency Analysis

**Figure 8:**
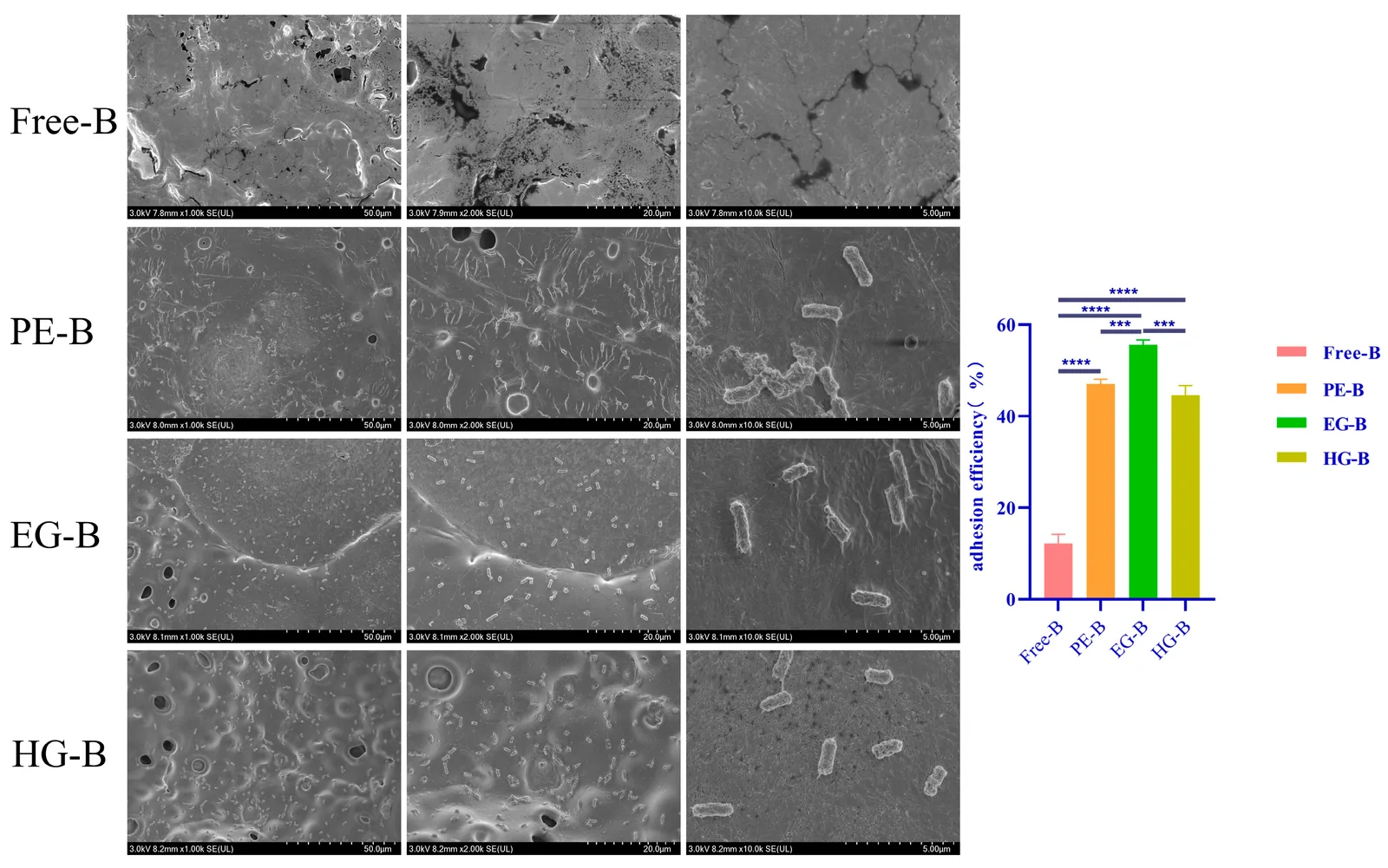
Adhesion efficiency (bar chart) of *Bifidobacterium longum* released from different delivery systems onto colonic tissue and corresponding Cryo-SEM micrographs. **** indicates *p* < 0.0001, *** indicates *p* < 0.001. (one-way ANOVA followed by Tukey’s multiple-comparison test). Data are presented as mean ± standard deviation (*n* = 3).

The colonic adhesion ability of *B. longum* in each system was assessed using an everted mouse colonic sac model, with results presented in Figure 8. The AE of Free-B was only 12.21%, while the efficiencies of PE-B, HG-B, and EG-B increased to 47.09%, 44.65%, and 55.64%, respectively, all significantly greater than that of Free-B (*p* < 0.0001). Among these groups, EG-B exhibited the highest AE, which was significantly superior to both PE-B and HG-B (*p* < 0.001).

The enhanced AE of EG-B can be attributed to its integrated oil-droplet-gel-network structure. This structure effectively maintains bacterial-cell viability after digestion, thereby indirectly preserving the integrity of bacterial surface adhesion-related functions [13]. Additionally, the inherent mucoadhesive properties of the calcium-alginate cross-linked gel network [46], combined with the hydrophobic interfaces provided by oil droplets, synergistically facilitated the adhesion of *B. longum* to the colonic epithelium [16,47]. Cryo-SEM observations supported these quantitative results: the colonic surface in the Free-B group displayed almost no visible bacterial cells, while rod-shaped *B. longum* were observed adhering to the tissue in the PE-B, HG-B, and EG-B groups. The EG-B group demonstrated the most abundant bacterial distribution with aggregated adhesion, visually confirming that this system promotes *B. longum* adhesion to the colonic mucosa.

No significant difference in AE was seen between PE-B and HG-B (47.09% vs. 44.65%, *p* > 0.05). While PE-B lacked a calcium-alginate cross-linked gel network, HG-B contained no oil phase. This finding indicated that the calcium-alginate cross-linked network and the oil-droplet interfacial layer exert comparable effects on bacterial adhesion when functioning independently. The strongest synergistic adhesion-promoting effect arose only when both components formed an integrated structure.

## 4. Conclusions

In this study, a structurally integrated emulsion gel (EG-B) was successfully developed via in situ calcium-ion crosslinking, utilizing TBP-SA nanocomplexes as both the stabilizer and gel precursor, thereby achieving efficient encapsulation and multi-level protection of *B. longum*. EG-B features a dual composite structure that includes a compact Pickering interfacial membrane and a continuous calcium-alginate gel network, endowing the system with advantageous rheological properties (low-shear viscosity of approximately 100 Pa·s), dilution stability, and shear adaptability. Under pasteurization conditions, EG-B maintained viable counts exceeding 6 log CFU/g, meeting the efficacy threshold for probiotic products. Gastrointestinal tolerance experiments demonstrated that the dual interfacial membrane-gel-network structure of EG-B provided a sequential protective effect against gastric acid and bile salts, with a final viable count of 6.12 log CFU/g significantly surpassing those of the single-structured PE-B and HG-B. After 14 days of storage at both 4 °C and 25 °C, EG-B consistently exhibited the highest retention of viability and demonstrated the strongest colonic adhesion capacity (55.64%) under a standardized initial bacterial-cell concentration.

The EG-B emulsion gel achieved simultaneous interfacial stabilization and gel network construction utilizing a single TBP-SA complex. It boasts a straightforward preparation protocol and consists of entirely food-grade ingredients, forming a probiotic delivery system that integrates processing adaptability, gastrointestinal tolerance, and storage stability. This study lays a theoretical foundation and offers technical references for applying protein–polysaccharide complex-based emulsion gels in functional foods.

The present study fabricated and evaluated EG-B in an in vitro model system, but several gaps remain before its practical application. First, real food matrices contain complex components such as proteins, dietary fiber, and salts that may interfere with TBP-SA adsorption and calcium cross-linking; validation in actual products such as yogurt and beverages is therefore required. Second, the high viscosity and gel-like texture of EG-B, along with its oil content, may affect sensory acceptance, while the compatibility of its flavor and mouthfeel with target products remains to be evaluated. Third, the laboratory-scale preparation (high-speed shearing and 12 h gelation at 4 °C) differs from continuous industrial production, and scale-up and cost assessment need to be addressed. Finally, this study examined only 14 days of storage, whereas commercial probiotic products typically require a shelf life of several months; long-term stability, particularly above 25 °C, and cold-chain dependence require systematic evaluation. Resolving these issues will facilitate the translation of TBP-SA emulsion gels from laboratory research to practical food applications.

## Data Availability

The original contributions presented in the study are included in the article, further inquiries can be directed to the corresponding authors.

## References

[1] McClements D.J. (2020). Nano-enabled personalized nutrition: Developing multicomponent-bioactive colloidal delivery systems. Adv. Colloid Interface Sci..

[2] Zhao R., Yu T., Li J., Niu R., Liu D., Wang W. (2024). Single-cell encapsulation systems for probiotic delivery: Armor probiotics. Adv. Colloid Interface Sci..

[3] Song L., Xu H., Chen C., Zhang Y., Zhang Z., Chen X., Liu S., Xiao H., Gu Q., Li P. (2025). Supramolecular self-assembly for probiotic delivery in food system: Responsive strategies, multifunctional carriers, future perspectives. Trends Food Sci. Technol..

[4] Hei X., Liu Z., Cao Y., Xu C., Ma J., Zhang S., Wang X., Xie N., Li X., Lv J. (2026). Polysaccharide-based intelligent responsive colloidal carriers for food bioactives: Structures, stimuli-triggered release mechanism and applications. Adv. Colloid Interface Sci..

[5] Edo G.I., Mafe A.N., Akpoghelie P.O., Gaaz T.S., Yousif E., Yusuf O.S., Isoje E.F., Igbuku U.A., Opiti R.A., Ayinla J.L. (2025). The utilization of biopolymer hydrogels to encapsulate and protect probiotics in foods. Process Biochem..

[6] Zang J., Yin Z., Ouyang H., Liu Y., Liu Z., Yin Z. (2025). Advances in the preparation, applications, challenges, and future trends of polysaccharide-based gels as food-grade delivery systems for probiotics: A review. Compr. Rev. Food Sci. Food Saf..

[7] Liu Y., Wang R., Lu Y., Zhang H., Yang Y., Feng X., Sun K., Ma L., Zhang Y. (2026). Modulating the properties of tuna skin collagen-based emulsion gels by moderate hydrolysis: Physical characteristics, stability, and lycopene delivery. Food Hydrocoll..

[8] Meng W., Miao S. (2026). Multifunctional emulsion gels for dysphagia nutrition: Coordinating texture, bioactives delivery, and sensory perception. Food Hydrocoll..

[9] An Y., Zhu Y., Xia X., Huang Y., Lv M., Zhu X. (2026). Structural properties, rheological characteristics, and drug delivery efficiency of resveratrol-loaded soybean protein isolate and hemp protein isolate emulsion gels: Synergistic effects of carboxymethyl cellulose incorporation and ultrasound treatment. Food Chem. X.

[10] Shu J., McClements D.J., Luo S., Liu C., Ye J. (2025). Advances of biopolymer-based emulsion gels: Fabrication, design, and application. Trends Food Sci. Technol..

[11] Song Y., Shen X., Zhou G., Xu X., Cao Y., Li W., Hu Y., Zhao J., Wu D., Huang Z. (2025). Fabrication and Mechanism of Pickering Emulsions Stability over a Broad pH Range Using Tartary Buckwheat Protein–Sodium Alginate Composite Particles. Foods.

[12] Xu D., Liu Z., An Z., Hu L., Li H., Mo H., Hati S. (2023). Incorporation of probiotics into 3D printed Pickering emulsion gel stabilized by tea protein/xanthan gum. Food Chem..

[13] Qin X., Bo Q., Qin P., Wang S., Liu K. (2023). Fabrication of WPI-EGCG covalent conjugates/gellan gum double network emulsion gels by duo-induction of GDL and CaCl_2_ for colon-controlled *Lactobacillus Plantarum* delivery. Food Chem..

[14] Wang K., Ni J., Tian X., Xiang S., Li H., Shang W., Liu B., Tan M., Su W. (2024). Survivability of probiotics in Pickering emulsion gels stabilized by salmon by-product protein / sodium alginate soluble complexes at neutral pH. Int. J. Biol. Macromol..

[15] Mao L., Pan Q., Yuan F., Gao Y. (2019). Formation of soy protein isolate-carrageenan complex coacervates for improved viability of Bifidobacterium longum during pasteurization and in vitro digestion. Food Chem..

[16] Wang L., Song M., Zhao Z., Chen X., Cai J., Cao Y., Xiao J. (2020). Lactobacillus acidophilus loaded pickering double emulsion with enhanced viability and colon-adhesion efficiency. LWT.

[17] Işçimen E.M. (2025). Production of a food-grade pickering emulsion stabilized by pea protein-different phenolic acids. J. Sci. Food Agric..

[18] Yin M., Zhang Q., Zhong F. (2024). Construction of double network gel for co-encapsulation of probiotics and capsaicin: Enhanced the physicochemical stability and controlled release. Food Biosci..

[19] Huang E., Li Z., Zhou F., Chen Q., He J., Xiong X., Xie Y., Gao H., Zou L. (2025). Encapsulation of *Lactiplantibacillus plantarum* subsp. *plantarum* in oil-phase gels O/W high internal phase emulsions: Improvement of storage stability and gastrointestinal viability. Food Chem..

[20] Chen X., Chen Y., Li J., Wang Y., Yang X. (2019). Enzyme-assisted development of biofunctional polyphenol-enriched buckwheat protein: Physicochemical properties, in vitro digestibility, and antioxidant activity. J. Sci. Food Agric..

[21] Han X., Nie Y., Wang T., Wang W., Yu D. (2026). Emulsion gel designed based on rice bran protein-pectin conjugated microgel particles: Interface characteristics, rheological properties and water migration. Carbohydr. Polym..

[22] Brodkorb A., Egger L., Alminger M., Alvito P., Assunção R., Ballance S., Bohn T., Bourlieu-Lacanal C., Boutrou R., Carrière F. (2019). INFOGEST static in vitro simulation of gastrointestinal food digestion. Nat. Protoc..

[23] Feng K., Huang R.-M., Wu R.-Q., Wei Y.-S., Zong M.-H., Linhardt R.J., Wu H. (2020). A novel route for double-layered encapsulation of probiotics with improved viability under adverse conditions. Food Chem..

[24] Abbasi S., Rafati A., Hosseini S.M.H., Roohinejad S., Hashemi S., Gahruie H.H., Rashidinejad A. (2023). The internal aqueous phase gelation improves the viability of probiotic cells in a double water/oil/water emulsion system. Food Sci. Nutr..

[25] Haji F., Cheon J., Baek J., Wang Q., Tam K.C. (2022). Application of Pickering emulsions in probiotic encapsulation—A review. Curr. Res. Food Sci..

[26] Bao S., Guo L., Xuan J., Yang L., Li Y., Jiang B., Ren D. (2025). Preparation of pinolenic acid-embedded *Lactiplantibacillus plantarum* H6 Pickering emulsions stabilized with different concentrations of sodium alginate. Food Biosci..

[27] Jo D.-M., Bae S.S., Chung D., Jung Y.-H., Jin H., Jeong H.G., Tabassum N., Kim Y.-M., Khan F. (2026). Hydrocolloid-based encapsulation systems for probiotics in foods: Structure–rheology–release relationships and rational design principles. Trends Food Sci. Technol..

[28] Cui X., Ma M., Xie Y., Yang Y., Li Q., Sun S., Ma W. (2022). Formation, structure and stability of high internal phase Pickering emulsions stabilized by BSPI-C3G covalent complexes. Food Chem. X.

[29] Varnaitė-Kapočė L., Kabalinaitė B., Petrikaitė V., Gölge E., Šipailienė A., Leskauskaitė D. (2025). Gelation of different phases of probiotic-loaded water-in-oil-in-water emulsion to enhance probiotic survival stability. Int. J. Food Sci. Technol..

[30] Lei L., Xu C.-H., Zhu C.-H., Zou Y.-C., Guo Y.-X., Bodjrenou M.D., Zhang B., Yuan Y. (2025). Flavor controlled release behavior from novel high internal phase Pickering emulsion gels stabilized by zein-chitin nanocrystals complex coacervates: The models on phase equilibria and mass transfer. Food Hydrocoll..

[31] Gao F., Wang X., Han X., Yu D., Wang W., Wang T. (2025). Incorporation of polysaccharides (κ-carrageenan and flaxseed gum) to improve the stability of rice bran oil body emulsion and oil body oleogel: Microstructure, physicochemical stability and digestive properties. Food Chem..

[32] Chen Z., Cui B., Guo X., Zhou B., Wang S., Pei Y., Li B., Liang H. (2022). Fabrication and characterization of Pickering emulsions stabilized by desalted duck egg white nanogels and sodium alginate. J. Sci. Food Agric..

[33] Yiu C.C., Wang Y., Selomulya C. (2025). Double Network as a Design Paradigm for Structuring Emulsion Gels in Food. Compr. Rev. Food Sci. Food Saf..

[34] Ručigaj A., Kopač T. (2025). Mathematical modeling of swelling and shrinking dynamics in pH-sensitive hydrogels composed of alginate, anionic cellulose, and chitosan. Polymer.

[35] Qiao L., Qin J., Liao H., Yang M. (2025). Characterization of a casein-sodium alginate emulsion gel. LWT.

[36] Zhang Q., Wang Y.-Q., Li L., Song H.-L., Wu H.-T., Zhu B.-W. (2024). Fabrication and characterization of salidroside W/O/W emulsion with sodium alginate. Food Chem. X.

[37] Keramat M., Kheynoor N., Golmakani M.-T. (2022). Oxidative stability of Pickering emulsions. Food Chem. X.

[38] Xu H., Chandrapala J., Dabbour M., Mintah B.K., Huang L., Dai C., Ma H., He R. (2025). Effect of xylose glycation and ultrasonication on the interfacial properties and physicochemical stability of silkworm pupa protein-stabilized Pickering emulsion and its applicability in emulsion-filled hydrogels. Food Res. Int..

[39] Chen Z., Gu Z., Cheng L., Hong Y. (2026). Starch-based emulsion gels enhanced probiotic viability: Effect of amylose content. Food Hydrocoll..

[40] Li X., Zheng K., Wu X., Zhu Z., Xu Z., Hou Z., Wang L. (2026). Pomelo fiber Pickering emulsion gels as light-cream fat mimetics in reduced-fat ice cream. Food Chem. X.

[41] Verheyen D., Govaert M., Seow T.K., Ruvina J., Mukherjee V., Baka M., Skåra T., Van Impe J.F.M. (2019). The Complex Effect of Food Matrix Fat Content on Thermal Inactivation of *Listeria monocytogenes*: Case Study in Emulsion and Gelled Emulsion Model Systems. Front. Microbiol..

[42] Wu N., Fan C., Qin G., Zhang X., Chen Z., Zhao M., Lu C. (2026). Characterization of Heat Conduction Performance in Sodium Polyacrylate Hydrogels with Varying Water Content. Materials.

[43] Huang L., Yang S., Bai M., Lin Y., Chen X., Li G., Cui L.-G., Wang X. (2022). Thermal shielding performance of self-healing hydrogel in tumor thermal ablation. Colloids Surf. B Biointerfaces.

[44] Liu W., Liu J., Salt L.J., Ridout M.J., Han J., Wilde P.J. (2019). Structural stability of liposome-stabilized oil-in-water pickering emulsions and their fate during in vitro digestion. Food Funct..

[45] Sarkar A., Zhang S., Holmes M., Ettelaie R. (2019). Colloidal aspects of digestion of Pickering emulsions: Experiments and theoretical models of lipid digestion kinetics. Adv. Colloid Interface Sci..

[46] Silvestre A.L.P., Viegas J., Carvalho A.M., Almeida H., Carvalho S.G., dos Santos A.M., Júnior A.G.T., Chorilli M., Sarmento B. (2026). Surface charge behavior of mezalazine-loaded chitosan–alginate nanoparticles in a 3D multi-layered intestinal in vitro model. Carbohydr. Polym..

[47] Alonso García E., de la Fuente Ordoñez J.J., Lavilla Lerma L., Estudillo-Martínez M.D., Castillo-Gutiérrez S., Benomar N., Knapp C.W., Abriouel H. (2021). Transcriptomic Profile and Probiotic Properties of *Lactiplantibacillus pentosus* Pre-adapted to Edible Oils. Front. Microbiol..

